# CBK model composition using paired web services and executable functions: A demonstration for individualizing preventive services

**DOI:** 10.1002/lrh2.10325

**Published:** 2022-08-03

**Authors:** Allen Flynn, Glen Taksler, Tanner Caverly, Adam Beck, Peter Boisvert, Philip Boonstra, Nate Gittlen, George Meng, Brooke Raths, Charles P. Friedman

**Affiliations:** ^1^ Department of Learning Health Sciences Medical School University of Michigan Ann Arbor Michigan USA; ^2^ Cleveland Clinic, Internal Medicine and Geriatrics Cleveland Ohio USA; ^3^ School of Public Health University of Michigan Ann Arbor Michigan USA

**Keywords:** computable biomedical knowledge, decentralized web technology, model composition

## Abstract

**Introduction:**

Learning health systems are challenged to combine computable biomedical knowledge (CBK) models. Using common technical capabilities of the World Wide Web (WWW), digital objects called Knowledge Objects, and a new pattern of activating CBK models brought forth here, we aim to show that it is possible to compose CBK models in more highly standardized and potentially easier, more useful ways.

**Methods:**

Using previously specified compound digital objects called Knowledge Objects, CBK models are packaged with metadata, API descriptions, and runtime requirements. Using open‐source runtimes and a tool we developed called the KGrid Activator, CBK models can be instantiated inside runtimes and made accessible via RESTful APIs by the KGrid Activator. The KGrid Activator then serves as a gateway and provides a means to interconnect CBK model outputs and inputs, thereby establishing a CBK model composition method.

**Results:**

To demonstrate our model composition method, we developed a complex composite CBK model from 42 CBK submodels. The resulting model called CM‐IPP is used to compute life‐gain estimates for individuals based their personal characteristics. Our result is an externalized, highly modularized CM‐IPP implementation that can be distributed and made runnable in any common server environment.

**Discussion:**

CBK model composition using compound digital objects and the distributed computing technologies is feasible. Our method of model composition might be usefully extended to bring about large ecosystems of distinct CBK models that can be fitted and re‐fitted in various ways to form new composites. Remaining challenges related to the design of composite models include identifying appropriate model boundaries and organizing submodels to separate computational concerns while optimizing reuse potential.

**Conclusion:**

Learning health systems need methods for combining CBK models from a variety of sources to create more complex and useful composite models. It is feasible to leverage Knowledge Objects and common API methods in combination to compose CBK models into complex composite models.

## 1 INTRODUCTION

In 2009, Tsafnat and Coiera discussed several challenges related to reasoning across multiple biomedical models.^1^ They highlighted the challenges of computer‐aided model construction, automated model selection, and model composition. This paper focuses on model composition, which is the process of building up better reasoning capabilities by connecting or combining multiple models to form *composite models*.^1^, ^2^

The topic of model composition is not new, but it is timely.^3^, ^4^, ^5^, ^6^ In biomedicine, there is growing evidence from fields including whole‐cell modeling and integrated systems biology that composite models can improve our understanding of biology and human health.^6^, ^7^ It is now conceivable that model composition could become central to a lot of future scientific work in biomedicine.^8^ Hence, for learning health systems, model composition seems vital.^9^

Throughout this paper, “model” refers to computer‐processable implementations of results and insights previously revealed through empirical scientific inquiry and learning. In these types of models, such results and insights are expressed concretely and formally as conceptual, logical, mathematical, or statistical statements about variables and the relationships between variables, including causal and correlative relationships.^10^

This paper contributes a new method for building composite models by connecting them via their inputs and outputs. We recognize that model composition in software is nothing new. Software that brings many models together by interrelating the inputs and outputs of discrete functions has been around for a long time. What is new here is that how the models we combine are individually externalized and modularized using compound digital objects called Knowledge Objects (KOs). We have previously published our Knowledge Object Reference Ontology (KORO) which describes the parts and pieces of Knowledge Objects in detail.^11^ Following KORO, the KOs for this study enable models to be treated both as static resources and active web services.

Using multiple KOs, we are primarily interested in composing models of *computable biomedical knowledge* (CBK). CBK models may also be called CDS artifacts or machine learning, deep learning, AI, decision, and business process models, or even *actionable knowledge units*.^12^, ^13^, ^14^ Here, we generally refer to any model that represents biomedical results and insights as a *CBK model*. To better support learning health systems, we demonstrate the building of *composite CBK models* by interconnecting multiple distinct CBK submodels packaged inside many individual KOs.

This model composition work is generally motivated by three main drivers relevant to learning health systems. First, the relational nature of knowledge calls for connections between disparate results and insights.^15^, ^16^, ^17^ Second, the acceleration of scientific activity and attendant accumulation of new results and insights increase the need to connect new and prior knowledge to extend and apply what is learned.^18^ Third, different types of knowledge exist and necessarily have dissimilar computer‐processable representations.^19^, ^20^, ^21^ Hence, to advance biomedical science, enable learning health systems, and improve human health, more distinct computable models represented variously need to be connected or combined effectively than in the past.^22^

Research and development on decentralized web technology for model reuse strongly influence our approach.^23^ Web app developers will be familiar with our approach since they are accustomed to building web applications with reusable *software libraries* or *packages*, some of which contain “models” per our definition of the term.^24^ Similarly, data scientists perform model composition using tools and languages that strongly support code reuse, such as Machine Learning in Julia (MLJ).^2^ In addition, to compute with models represented variously, polyglot virtual machines supporting a wide variety of *runtimes* (or software execution engines) enable model composition of submodels encoded in different programming languages and formats.^25^

Our CBK model composition method ultimately relies on connecting pairs of web services and corresponding executable functions backing the web services. We apply our method to connect and combine preventive medicine models into a composite model with 42 submodels to support *individualized precision prevention* (IPP). In the end, the composite model we produce computes individualized estimates of life gain for 21 different evidence‐based preventive services.^26^

Next, in the Methods section, we begin by outlining our general technical approach to model composition. After that, we detail key technical items and give examples. Later, in the Results section, we describe the IPP composite model we developed with our methods and explain its use in an initial study of preventive service practices. Finally, we discuss our progress in the Discussion section and reflect on some key remaining challenges before concluding.

## 2 METHODS

### 2.1 GENERAL TECHNICAL APPROACH TO MODEL COMPOSITION

**FIGURE 1:**
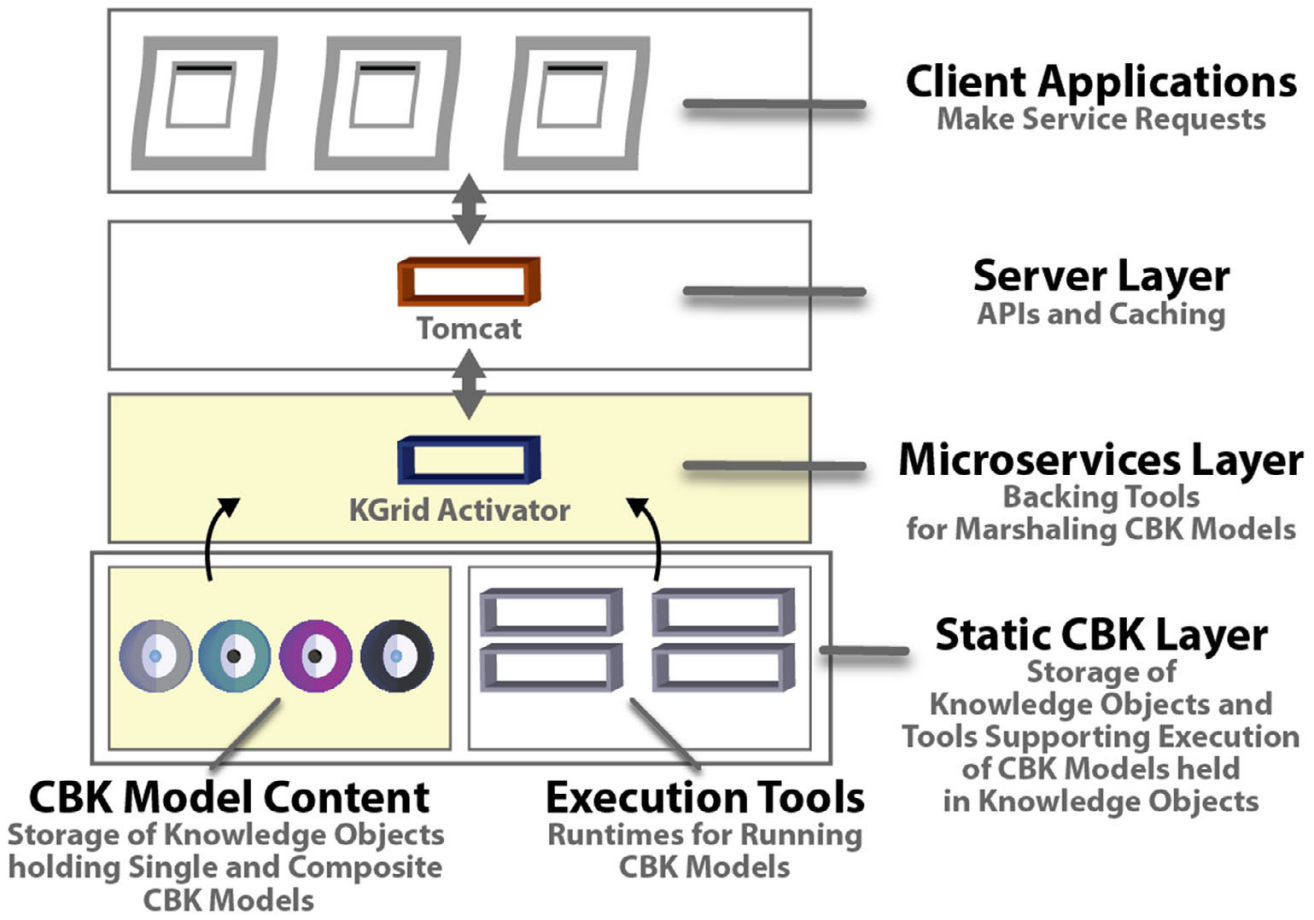
Technical stack enabling model composition

Our primary goal is to support both the developers and end‐users of end‐user or *client applications* by giving developers and end‐users ready access to powerful composite CBK models. As portrayed in Figure 1 below, our approach uses a stack of technical components for managing and deploying KOs, which are digital packages holding CBK models.^27^ In Figure 1, the two yellow‐shaded areas are where we make new technical contributions.

In the Server Layer of our technology stack just below the client applications layer, we rely on established World Wide Web (WWW) network components to handle standard HTTP requests and responses to and from lower‐level microservices. In the Microservices Layer, custom microservice tools from our team, especially the Knowledge Grid (KGrid) Activator,^27^ organize, mobilize, and instantiate CBK models to get them running and make them network accessible.

In the lowest static CBK Layer of our stack, we specify the structure and contents of modular Knowledge Objects.^11^, ^28^ Each KO is stored as an individually identifiable package that bundles a distinct CBK model along with some other essential content described below. KOs can then be used for computing with assistance from the KGrid Activator tool.

Our stack brings in well‐supported runtimes built by others for executing the CBK models packaged in KOs (Figure 1). To date, we have used runtimes for JavaScript (eg, V8), Python, and R. Theoretically, there is no limit to the number of runtimes that can be incorporated in our technical infrastructure. Therefore, CBK models encoded in almost any programming language or format can be deployed and connected to form composites thereby extending our approach.

Next, we cover more about our approach to CBK model composition. The first key item we review in further detail is the Knowledge Object or KO packages holding CBK models (Figure 1).

### 2.2 KNOWLEDGE OBJECTS AND THEIR CONTENTS

**FIGURE 2:**
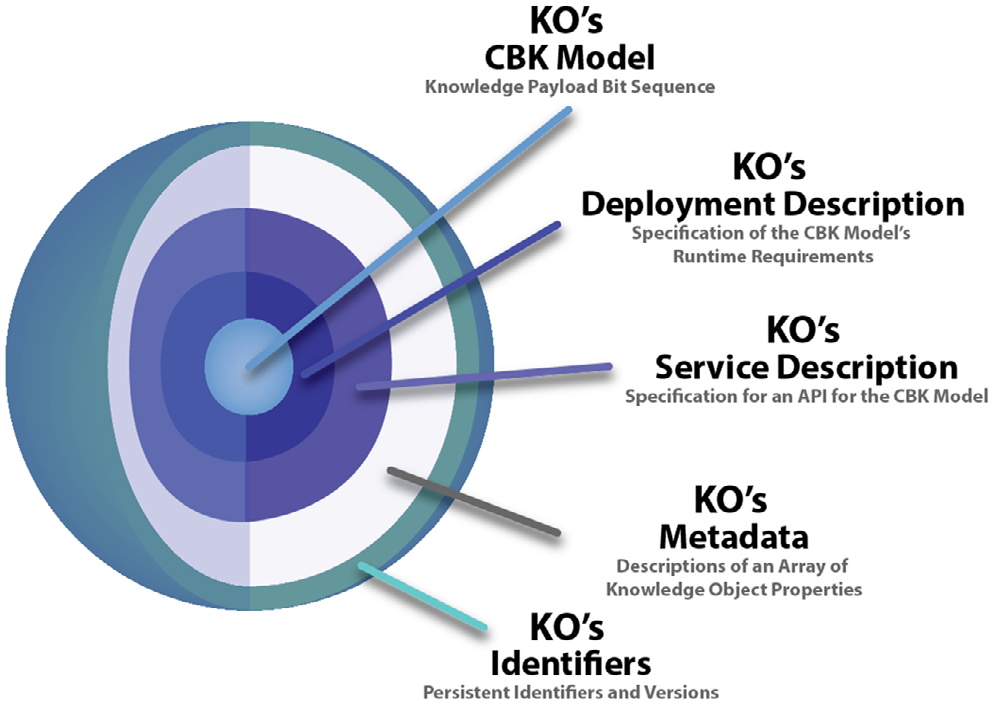
Contents of a knowledge object (KO),^11^ a particular subclass of compound digital objects^28^

Our approach to CBK model management begins with formalized Digital Objects (DOs).^28^ All DOs have three things, (a) a *bit sequence* expressing some core content, (b) *metadata* describing object properties, and (c) a *persistent unique identifier*.^28^ We previously specified the new class of DOs called Knowledge Objects (KOs) diagrammed in Figure 2 below.^11^

KOs provide the means to manage CBK models both as static *resources* and to deploy CBK models as *web services*. As static resources, CBK model creators and owners can place their models inside KOs as files and then transmit and share KOs over computer networks, organize KOs in digital repositories, build collections and libraries of KOs, and archive KOs for long‐term safekeeping.^29^ When deployed as web services, CBK models can be engaged via APIs.^23^, ^27^

We have previously shown how CBK model deployers can use KOs with the tools we have built to instantiate web services quickly and systematically.^30^ The web services that result offer persistent, interactive, remote computational capabilities. These web services can also provide a mechanism to distribute CBK models directly to client applications on demand. Some of the ancillary content inside KOs exists to help establish web services for computing with CBK models.

We continue exploring how best to construct easily deployable, interoperable KOs that meet the needs of multiple CBK model stakeholders, including CBK model creators, owners, organizers, deployers, and of course client application developers and users. Recently, some of our team members have closely examined the metadata needed to make large numbers of CBK models packaged in compound digital objects findable, accessible, interoperable, and reusable (the FAIR principles^31^, ^32^). Next, following from the innermost to outermost components shown in Figure 2 above, we give examples of the information content for each component of the generic KO.

### 2.3 EXAMPLE OF A SIMPLE CBK MODEL HELD INSIDE A KNOWLEDGE OBJECT

We created a KO that holds a simple CBK model (Box 1). This model uses JavaScript code to relate two variables in a common formula for body mass index (BMI). The code shown is a computer‐executable representation of the mathematical function for BMI (Box 2).

BOX 1 **Body mass function encoded in the JavaScript programming language**

**function bmi(inputs){**


 **height = inputs.features.height; // height in inches**


 **weight = inputs.features.weight; // weight in pounds**


 return weight/height/height*703;

**}**

BOX 2 **Body mass index formula**

$\mathrm{Body Mass Index}\;\left(\mathrm{BMI}\right)=\frac{\frac{\text{bodyweight}\left(\mathrm{lb}\right)}{\text{height}\left(\text{in}\right)}}{\text{height}\left(\text{in}\right)}$ * 703

The purpose of a CBK model expressing the BMI formula (Box 1) is to compute an index for body weight relative to height. Originally dubbed the Quetelet Index after its inventor and later labeled the Body Mass Index (BMI) by Keys, when concretized in code, this simple CBK model plays an ongoing role in biomedical research and practice.^33^ While it makes a good example, the simplicity of this CBK model for BMI is misleading since CBK models are often much more complex.

We currently limit the CBK model content held in Knowledge Objects to explicit instances of executable code or machine‐readable data and do not support Knowledge Objects containing pointers to CBK models kept elsewhere. This limitation reflects the high priority we give to making CBK models accessible and their use secure by running them nearby protected health data sources. We recognize other CBK model use cases may be better supported by using pointers.

### 2.4 EXAMPLE OF THE DEPLOYMENT DESCRIPTION CONTENT INSIDE A KNOWLEDGE OBJECT

Every KO carries a Deployment Description file rendered in a simple format that we devised (Box 3). Deployment Description files convey a small amount of critical content. For example, the Deployment Description given in Box 3 below specifies a suitable runtime for executing the CBK models packaged in KOs (engine:node), the name of an executable file to be used as an entry point (“bmi.js”), a list of executable artifacts (here there is only one, bmi.js), and the name of an instantiable function for computing body mass index (function: bmi).

BOX 3 **Example of actual deployment description file content**

**/bmi:**


 **post:**


 **engine: node**


 **entry: bmi.js**


 **artifact: bmi.js**


 **function: bmi**

To improve standardization, we are exploring possible conventions for representing runtime information.^34^ Also, our work is so far limited to CBK models that are pure, stateless executable functions, like BMI. Pure functions associate one or more inputs to a single output.^34^ Pure functions make no changes to variables outside of the function's scope, warding off software side effects. In the future, we plan to extend our work to cover stateful CBK models too.

### 2.5 EXAMPLE OF THE SERVICE DESCRIPTION CONTENT INSIDE A KNOWLEDGE OBJECT

Alongside CBK models, KOs hold Service Description files. These files specify an application programming interface (API) for each web service associated with a CBK model. We currently render API specifications in the machine‐readable Open API 3.0 format for RESTful web service APIs.^35^ Other formats could be used, such as AsyncApi 2.0 for event‐driven web service APIs.^36^

A snippet from an actual Service Description file in the Open API 3.0 format is provided in Box 4 below. The version of the web service version (1.0) is different from versions of the CBK model or versions of the whole KO, which appear elsewhere in our metadata. Looking at the content of the Service Description, when put together with a deployed server's IP address (not shown), the partial URL given (/ipp/bmicalculator/1.0) and the path specification (/bmi) comprise a reachable URL access API endpoint that a developer can use to engage the BMI CBK model as a webservice.

BOX 4 **Snippet showing content from an actual service description file**

**openapi: 3.0.0**

**info:**


 **version: '1.0'**


 **title: BMI Calc**


 **description: Calculates BMI**


 **license:**


 **name: GNU General Public License v3 (GPL‐3)**


 **url: >−‐**


 **https://tldrlegal.com/license/gnu‐general‐public‐license‐v3‐(gpl‐3)#fulltext**


 **contact:**


 **name: KGrid Team.**


 **email:** 
kgrid-developers@umich.edu



 **url: 'http://kgrid.org'**

**servers:**


 **‐ url: /ipp/bmicalculator/1.0**


 **description: BMI Calculator**

**tags:**


 **‐ name: BMI Calculator Endpoint**

**paths:**


 **/bmi:**

### 2.6 OTHER CONTENT INSIDE A KNOWLEDGE OBJECT

We have covered the CBK model, Service Description, and Deployment Description packaged inside of KOs. In addition to this content, KOs also contain a metadata file with a linked data representation of the title, authors or owners, and KO version. Inside our metadata files, one also finds the persistent unique identifier (PUID) for the KO. This PUID, along with the rest of the KO metadata, supports the search and discovery of large numbers of uniquely identified KOs.

Next, in support of our general technical approach, we describe the KGrid Activator we have built. This tool sits in the Microservices Layer of our technical stack (Figure 1).

### 2.7 THE KGRID ACTIVATOR MICROSERVICE TOOL

To enable our model composition method, we designed and developed the KGrid Activator. The KGrid Activator is a server‐side backend tool that apps can communicate with. It is built as a Java microservice tool^37^ with the help of the Java Spring Framework.^38^ The KGrid Activator is KO‐aware. It activates KOs and then serves as an API gateway, orchestrating the execution of the CBK models packaged in KOs.^27^ The KGrid Activator is a *reference implementation* of a backend tool. It enables us to continuously test our commitment that all models held in KOs will run.

To assist CBK model deployers, the KGrid Activator implements a repeatable pattern to “activate” CBK models. In this case, activation of CBK models is the rapid and consistent deployment of web services backed by running CBK models. To demonstrate the feasibility of our approach to technical experts who might be tasked with deploying web services backed by CBK models, we set an initial performance benchmark to “activate” CBK models held inside KOs in 5 seconds or less by giving simple commands to the KGrid Activator. A 5 second time‐to‐deployment is in keeping with the time required by other container‐based deployment infrastructures like Docker.

**FIGURE 3:**
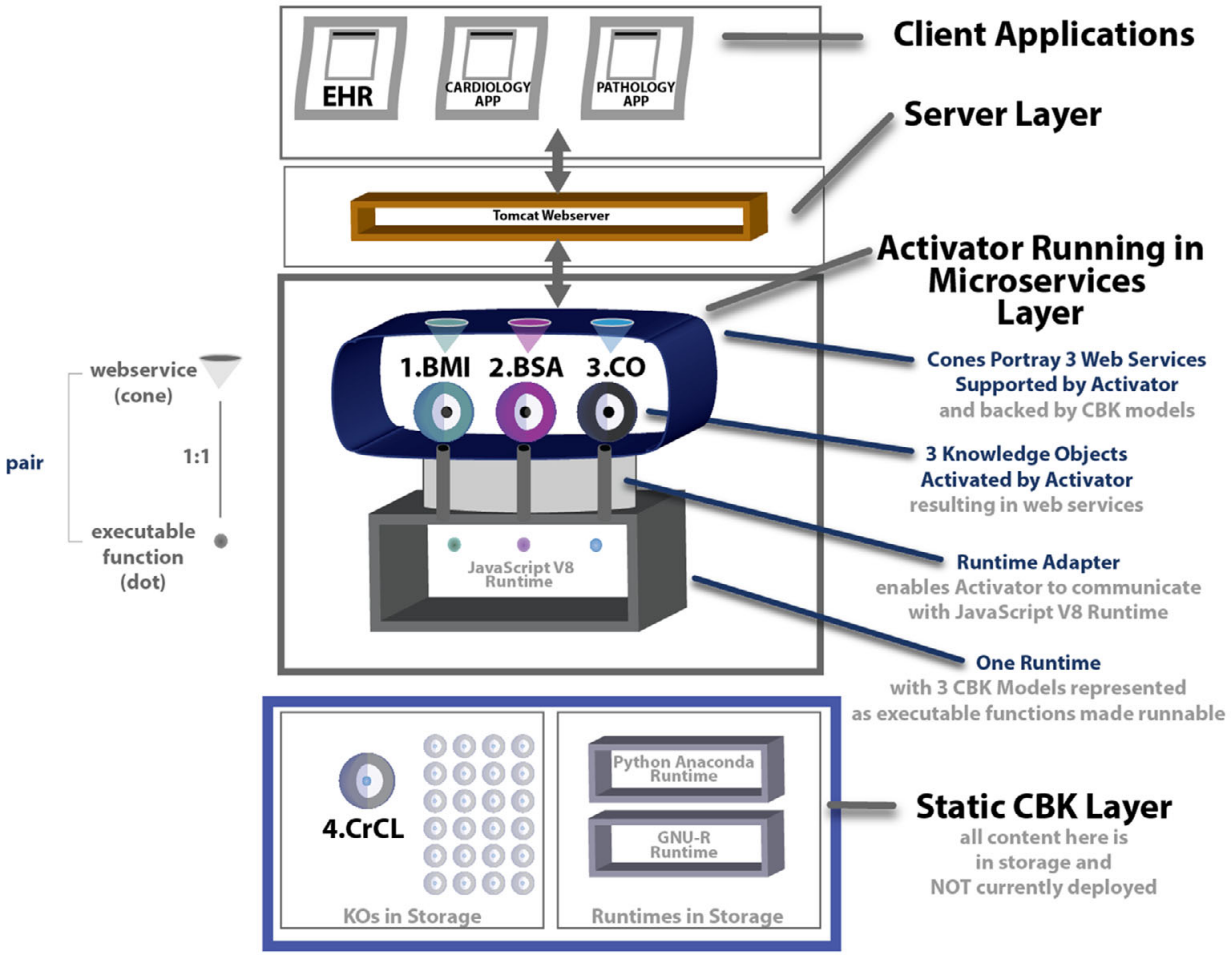
View of the KGrid activator operating in the microservices layer

Figure 3 below portrays how the KGrid Activator works to support one or more end‐user client applications with web services backed by CBK models. Starting at the top of Figure 3, three client applications are shown, an EHR next to a cardiology and pathology app. These and other apps (including SMART apps and CDS Hooks) can be programmed to engage executable CBK models via calls to typical web servers (eg, Tomcat) that in turn shuttle information to and from the web services enabled by the Activator.

Figure 3 includes four very simple example CBK models encoded in JavaScript. The first CBK model is the BMI model described previously (1.BMI). The second is a formula for body surface area (2.BSA). The third is a formula for computing cardiac output using stroke volume and heart rate (3.CO). The fourth is a formula for calculating creatinine clearance by the kidneys (4.CrCL). In Figure 3, the first three models have been deployed and activated by an instance of the Activator, giving rise to three pairs of corresponding web services (cones) and deployed executables (dots). Once activated, the deployed executables (dots) are shown running on demand inside an instance of the JavaScript V8 runtime. Note that the fourth model (4.CrCL) is held inside a KO at the bottom of Figure 3 alongside what could potentially be many other KOs that are in storage but ready to be activated and used anytime. These stored KOs are not currently deployed.

Moving further down the technology stack depicted in Figure 3, the KGrid Activator activates KOs with the help of a specific Runtime Adapter that we have built. This adapter allows the KGrid Activator to communicate with the JavaScript V8 runtime. Using it, the Activator can command the JavaScript V8 runtime to accept and run CBK models 1, 2, and 3, as shown. Finally, on the bottom right of Figure 3, two other runtimes, Python Anaconda and GNU‐R, are portrayed. These two additional runtimes have not yet been instantiated and connected to the Activator, although they could be if KOs with CBK models encoded in Python or R needed to be activated as well.

Summarizing, in Figure 3, activation of three CBK models for body mass index (BMI), body surface area (BSA), and cardiac output (CO) establishes three corresponding web services (green, pink, and blue cones). After the KGrid Activator provides web service endpoints to the Server Layer, it plays an API gateway role by routing incoming requests from external Client Applications to the CBK models running as executable functions inside one or more deployed runtimes. Now that we have covered the content of KOs, activation and the KGrid Activator, the last item to introduce as part of our technical methods is the *Runtime Context*.

### 2.8 HOW A RUNTIME CONTEXT ENABLES CBK MODEL COMPOSITION

**FIGURE 4:**
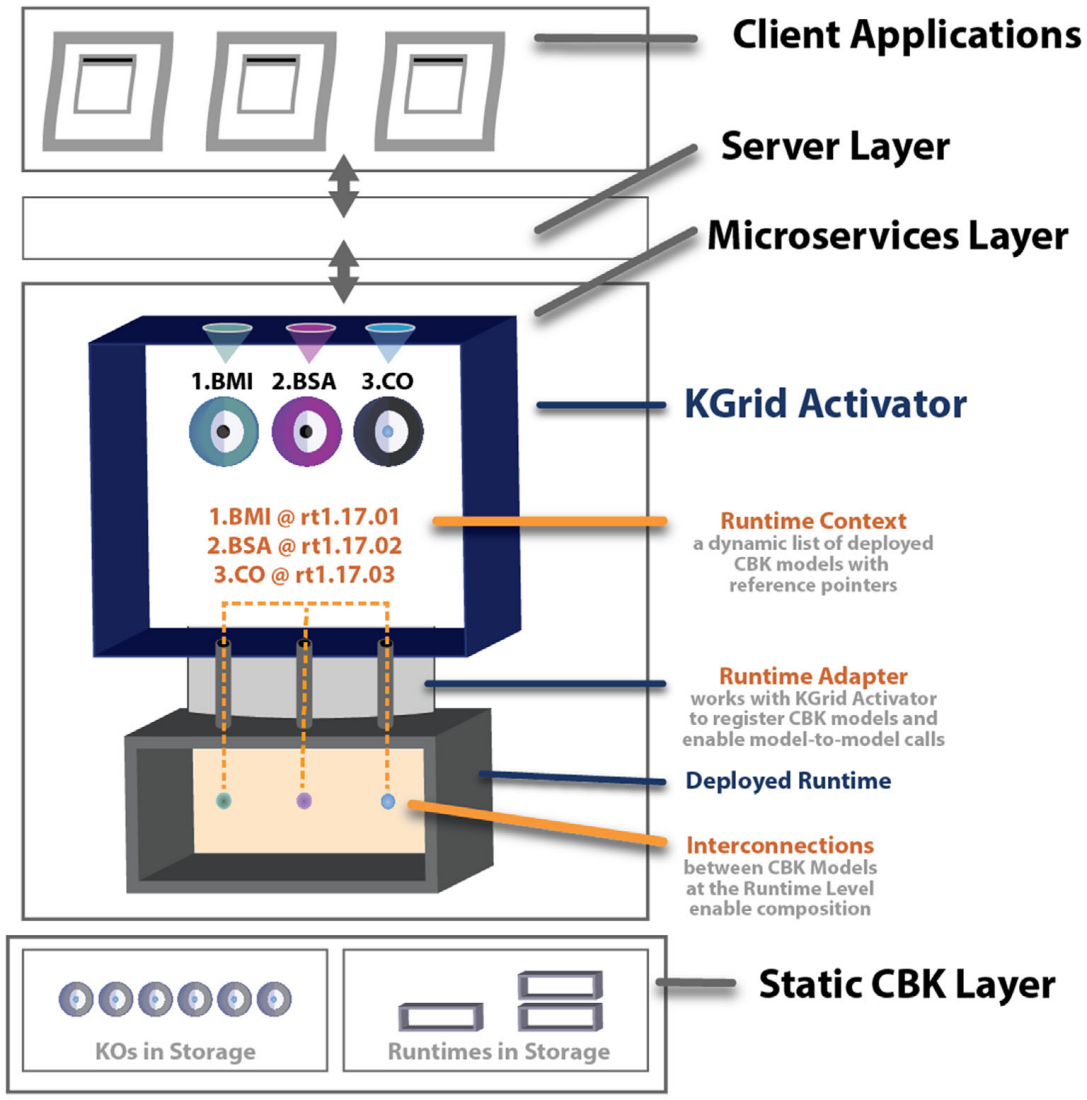
Inside the KGrid activator, the runtime context is a dynamic list of CBK models running locally

As part of the work of activating CBK models held inside KOs, each instance of the KGrid Activator establishes and maintains a dynamic list of every CBK model it has deployed with an internal reference pointer to each deployed CBK model (Figure 4). This list is the *Runtime Context*.

The Runtime Context provided by the KGrid Activator is accessible programmatically. For our method of model composition, when a request from the code of one CBK model is made to use another CBK model the KGrid Activator uses its current Runtime Context to operate as a gateway. In its gateway role, the KGrid Activator resolves CBK model references and oversees request‐response execution. This is how one deployed CBK models can call on any other deployed CBK model without having to know its implementation or instantiation details (Figure 4).

Going deeper into how this works, complete resolution of the reference pointers to deployed CBK models is achieved by the KGrid Activator working in conjunction with each runtime. This is where our runtime adapters come into play. We provide adapters to handle programming language and runtime‐specific concerns. (As one example, an adapter for the JavaScript V8 runtime is depicted in Figure 3.) Each adapter defines and implements a common communication protocol (ie, interface) we developed to integrate different runtimes with the KGrid Activator. Upon successful deployment of a CBK model into its corresponding runtime by the KGrid Activator, the model is registered by the adapter resulting in a new entry to the KGrid Activator's current Runtime Context. As a result, working in conjunction with the KGrid Activator, adapters allow CBK model‐to‐model request‐response activity to finally happen.

The Runtime Context is critical for interconnecting models and enabling model composition. For now, we have this capability only when every CBK model being connected is deployed inside the same runtime. We see a need for flexibility and power and plan to develop new capabilities so that future model composition can span multiple runtimes with the help of an enhanced KGrid Activator. Once additional CBK model‐to‐model request‐response capabilities are developed, KOs handling separate parts of the complex calculations can be deployed in separate runtimes. This will make it possible to build composite CBK models using component CBK models that are encoded in more than one format or programming language.

To summarize, as portrayed in Figure 4 below, the Runtime Context is a dynamic list or map maintained by an instance of the KGrid Activator. The Runtime Context supports all possible request‐response calls between activated CBK models running inside one runtime instance at any moment in time. As the number of CBK models deployed into that runtime instance changes, the Runtime Context is consistently refreshed and remains current. This approach is what finally enables our technology stack to support CBK model composition.

### 2.9 CREATING COMPOSITE CBK MODELS

**FIGURE 5:**
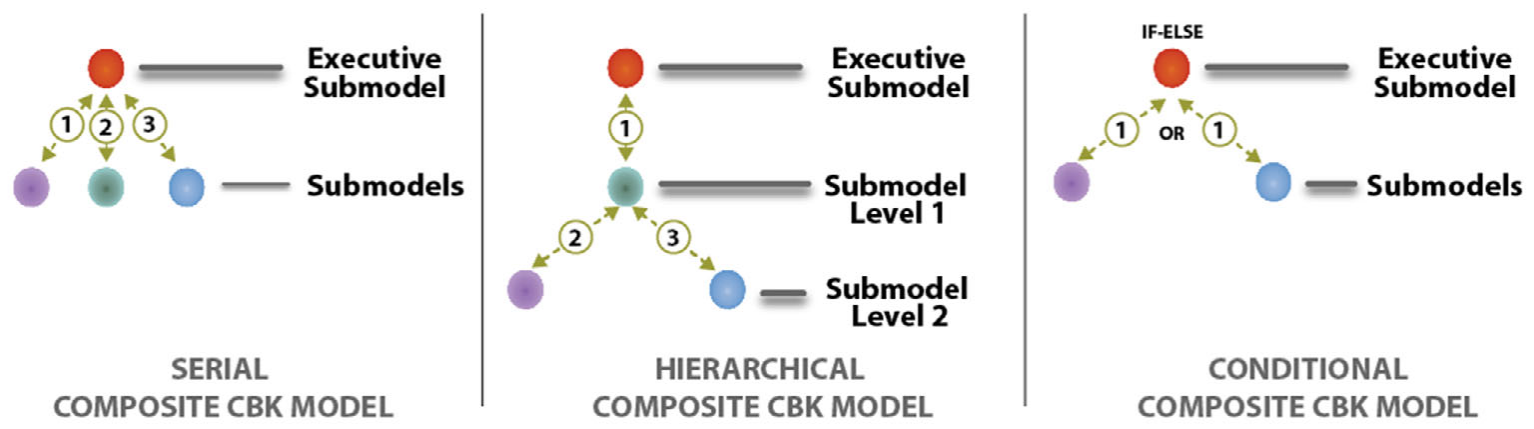
Three general kinds of composite CBK models

Instead of building all new technology for doing model composition, the KGrid Activator marshals the specific contents of KOs and then uses existing web server and runtime technologies for model composition. Together, the modular components in our technology stack are sufficient to create three general kinds of composite models (Figure 5). These three kinds of composite models ‐ serial, hierarchical, and conditional ‐ derive from a larger collection of common computational workflow patterns that have appeared repeatedly over the years in many other computer science works.^39^

On the left in Figure 5, a *serial composite CBK model* relies on the procedural knowledge about a model composition represented in an Executive Submodel (orange dot) to engage three submodels sequentially in steps 1, 2, and 3.

Similarly, in the middle of Figure 5, a *hierarchical composite CBK model* has a top‐level Executive Submodel connected to another Executive Submodel at Level 1 (green dot), which in turn encodes procedures to connect the two other submodels at Level 2 (purple and blue dots).

Additionally, it is possible to insert conditional “IF‐THEN‐ELSE” logic into composite models. As an example of this, on the right in Figure 5 is a *conditional composite CBK model* with an Executive Submodel that, depending on some condition, connects to one or the other (but not both) of the two submodels shown (purple and blue dots). The procedural knowledge encoded into the logic of Executive Submodels expresses model compositions. All three general kinds of composite models in Figure 5 ‐ and mixtures of them ‐ can be composed using our technical approach.

### 2.10 TECHNICAL FEASIBILITY DEMONSTRATION: BUILDING AND USING THE CM‐IPP

As a demonstration of the feasibility of our technical approach to model composition, we created the *Composite Model for Individualized Precision Prevention* (CM‐IPP). We used the CM‐IPP to conduct a concordance study which we will report elsewhere. (The concordance study sought to answer the research question. “To what degree do primary care providers collectively agree with the value‐based rankings of 21 preventive services computed by the CM‐IPP?”)

According to the United States Preventive Services Task Force (USPSTF), the 21 preventive services covered by the CM‐IPP are supported by high‐quality “A” or “B” level evidence. Examples of these services include screening services (eg, screening for colorectal cancer) and other measures (eg, taking aspirin to lower the risk of a heart attack). Studies show that in the U.S., many people receive some recommended preventive services, but few receive them all.^26^ The ultimate purpose of composite models like the CM‐IPP is to enable computerized individualized prioritization of recommended preventive services during routine primary care encounters. Next, for our results, we provide a detailed technical description of the CM‐IPP.

## 3 RESULTS

We successfully re‐implemented a multi‐component statistical model created and originally implemented as a Markov Cohort model inside a spreadsheet by Taksler and colleagues as part of their work in preventive medicine.^26^ Working with Dr. Taksler and his group, using KOs and our technical approach, we arrived at the new CM‐IPP composite CBK model for computing an individual's life‐gain arising for 21 recommended preventive medical services. When client applications engage the new CM‐IPP, the applications receive a ranked list of relevant preventive services for an individual. The CM‐IPP applies static and simplified results of Markov Cohort modeling to compute estimated life‐gain from regression formulas where some Markov Cohort model information is lost. In the future, the Taksler research team plans to use microsimulation modeling techniques^40^ and provide results either as lookup tables or mathematical equations.

### 3.1 CM‐IPP INPUTS AND OUTPUTS

The input to the CM‐IPP includes more than 100 features about a person. Any client application with these input data can post an instance of our proprietary JSON data object (Box 5) to the web service backed by the CM‐IPP's top‐level Executive Submodel. Client applications receive computed rankings of 21 relevant preventive services by estimated lifespan gain in years (Box 6).

BOX 5 **Partial list of inputs for the CM‐IPP web service**

**INPUT** {



 "patient":{



 "id": "ipp‐patient‐E01",



 "features":{



 "age":50,



 "race":"Black",



 "gender":"Male",



 "height":73.0,



 "weight":220.0,



 "systolic":147,



 "diastolic":68,



 "totalcholesterol":110,



 "HDL":32,



 "LDL":42,



 "triglycerides":181,



 "a1c":9,



 "cvd":false,



 [ *88 more features listed as key:value pairs go here* ]

BOX 6 **Partial list of outputs provided by the CM‐IPP web service**

**OUTPUT**"lifeexpectancy": {



 "aspirinPrevention": {



 "total": {



 "life‐gain": 0.3175741769690106 }



 },



 "crcScreening": {



 "total": {



 "life‐gain": 0.0919283879684265 }



 },



 "diabetesControl": {



 "total": {



 "life‐gain": 1.5806718157369808 }



 },



 [ *more computed results go here* ]

### 3.2 MORE TECHNICAL DETAILS ABOUT THE CM‐IPP

**FIGURE 6:**
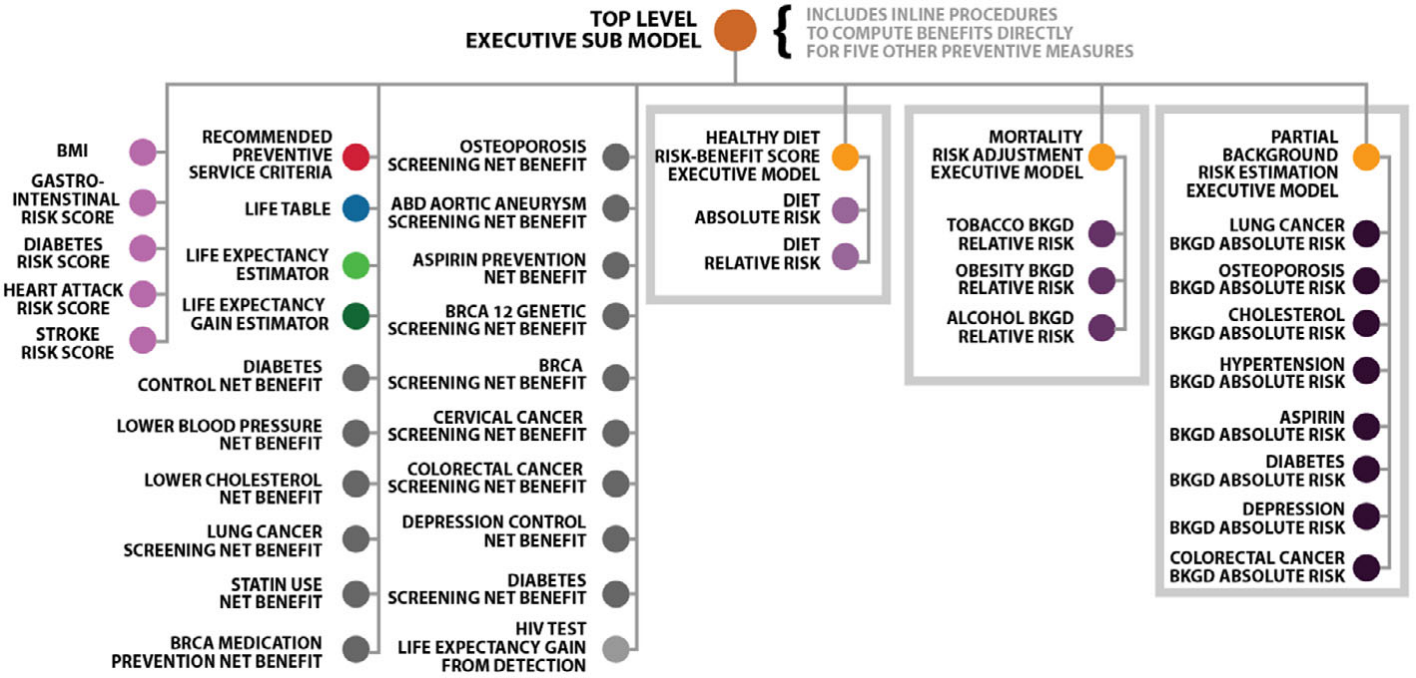
Graphical view of the CBK composite model for individualized precision prevention (CM‐IPP)

The CM‐IPP is a mixed *serial‐hierarchical‐conditional* composite model that performs a long and complex call chain of computations about an individual. This complex call chain (not shown) takes advantage of the 42 submodels of the CM‐IPP that are arrayed graphically in Figure 6 below.

As depicted in Figure 6, the CM‐IPP's top‐level Executive Submodel calls on 29 other submodels directly, three of which are lower‐level Executive Submodels (orange dots). The three lower‐level Executive Submodels call on a total of 13 more submodels. Conditional logic inside the top‐level Executive Submodel makes it so that only the relevant Net Benefit submodels (gray dots) are engaged while doing computations about a given person. In addition, the top‐level Executive Submodel includes more computable knowledge to compute benefit estimates for five preventive services directly.

Overall, we used 11 different types of submodels to build the CM‐IPP. Table 1 has descriptions of each of the 11 submodel types that contribute to the overall CM‐IPP.

**TABLE 1:** Eleven types of submodels connected to create the CBK composite model for IPP (CM‐IPP)

Submodel type	Description of submodel type	Quantity in CBK‐CM‐IPP
Executive	These include one top‐level and three lower‐level mostly procedural submodels with logic representing model composites. The web service backed by the top‐level Executive model accepts a large data object as its input from an external Client Application to kickoff CM‐IPP processing. The three lower‐level executive submodels compute various intermediate results with the help of other submodels.	4
Patient feature derivation	These submodels further process original patient data input to derive additional patient features (ie, BMI and common risk scores).	5
Recommended preventive service criteria	This submodel encodes inclusion and exclusion criteria, including age ranges, sex, and other criteria for all included preventive medical services covered in the CM‐IPP.	1
Life table	This submodel contains final numeric results for baseline mortality rates by age, race, and sex from the US Centers for Disease Control and Prevention.	1
Life expectancy estimator	This submodel calculates an individual's life expectancy using a given age range and a previously computed set of age‐ordered mortality rates.	1
Life expectancy gain estimator	This submodel accepts two life expectancy estimates computed with the Life Expectancy Estimator submodel as inputs and computes their difference.	1
Net benefit estimators	These submodels compute the estimated net benefit in terms of marginal life‐gain arising from implementing most preventive services and related interventions.	15
Detection gain estimator	This submodel calculates an individual's life expectancy gain from detection of HIV through routine testing.	1
Diet risk models	These two submodels estimate relative and absolute risks related to maintaining or not maintaining a healthy diet.	2
Mortality risk adjustment	These three submodels support computed adjustments in all‐cause mortality risk based on an individual's degree of obesity and alcohol and tobacco use.	3
Partial background risk estimation	These submodels compute background absolute risk for several diseases (eg, lung cancer) and several treatments or interventions (eg, taking aspirin.)	8

### 3.3 USING THE CM‐IPP IN PREVENTIVE SERVICES RESEARCH

Table 2 summarizes the end results of computations made using the CM‐IPP for 12 fictitious but realistic patient cases. These cases are part of a research study about preventive services.

**TABLE 2:** For each fictitious case, the Top 3 ranked services computed by the CM‐IPP are listed

Fictitious patient case	Source of ranking	Top‐ranked service	Second‐ranked service	Third‐ranked service	Fictitious patient case	Source of ranking	Top‐ranked service	Second‐ranked service	Third‐ranked service
#4	CM‐IPP	BRE	CRC	DIET	#2	CM‐IPP	LUN	STA	DIET
#6	CM‐IPP	BP	SMO	ALC	#7	CM‐IPP	CRC	DIET	AAA
#5	CM‐IPP	SMO	BP	DIET	#8	CM‐IPP	DIET	WEI	LUN
#11	CM‐IPP	SMO	DIET	BP	#10	CM‐IPP	BRE	CRC	DIET
#1	CM‐IPP	DIET	CRC	ASA	#12	CM‐IPP	DIET	ALC	SMO
#3	CM‐IPP	ALC	DIA	BP	#9	CM‐IPP	ALC	DIET	WEI

*Note*: The letter codes stand for abdominal aortic aneurysm screening (AAA), reducing alcohol use (ALC), aspirin use (ASA), treating blood pressure (BP), breast cancer screening (BRE), colorectal cancer screening (CRC), treating diabetes (DIA), diet counseling (DIET), statin use for cholesterol (STA), smoking cessation (SMO), and losing 10 pounds of weight (WEI).

## 4 DISCUSSION

We set out to compose distinct biomedical models using a repeatable and transferable method and achieved our goal. We built the CM‐IPP, a composite model that reasons and computes with 42 submodels representing biomedical knowledge from different sources (eg, AHRQ and CDC).

Our model composition method makes a strong commitment to decentralized web technology. For client applications that can access the web, our method takes advantage of HTTP server technology, common web service architectural patterns, and publicly available runtimes, such as JavaScript V8. Thus, our work aligns well with that of the WWW community and its massive installed base.

On the plus side, much of the software in this installed base interoperates and scales well already. However, we also inherit many challenges with the WWW. First among them, to make good on our approach, we must gain widespread adoption of new model packaging and activation specifications implemented using Knowledge Objects and the KGrid Activator, respectively. Second, all WWW security issues apply. At every level of our technology stack, the implementation of security protocols is required to protect CBK model stakeholders from harm. Third, many issues remain with measuring and expressing the quality of CBK models. We have demonstrated how to compose CBK models but not how to ensure the models composed are safe and effective for their intended use. In our future work, we anticipate using more than one evidence‐based composite model to generate advice and then using other composite models to prioritize competing and conflicting advice based on evidence grades and other relevant factors.

In this initial work, we have achieved a measure of *syntactic interoperability* via RESTful APIs but have yet to work on *semantic interoperability* for the inputs and outputs of CBK models.^41^ We have also packaged the CM‐IPP so that client application developers can build it directly into a code base instead of engaging it as a web service.

For composite model design, our work highlights the need for more and better principles to guide submodel modularization and composition. We struggled with issues of submodel scope and performance. For example, the top‐level Executive Submodel in the CM‐IPP mixes overarching procedural code for model composition with domain‐specific logic for five preventive services, making it more difficult to reuse this submodel in other contexts. Also, in hindsight, the 15 submodels used for net benefit estimation might have built into a single submodel instead.

Our experience does at least suggest two principles to inform decisions about submodel scope. First, a distinct submodel may be justified when it is subject to very frequent updates. Second, a distinct submodel may be justified if it can be reused in many composite models. Otherwise, the extra work of packaging and managing a distinct submodel seems not to be worth it.

Our work on model composition raises important knowledge management issues too. One of them is versioning many items, including the parts of KOs and model subcomponents, composite models, and the web services backed by such models. Good dependency management relies on item‐level versioning. We wish to extend our version‐control capabilities by applying lessons learned by those who have used software package metadata, automation, and human workflows to keep up with direct and transitive dependencies in decentralized web systems.

Another knowledge management challenge is how to categorize different types of submodels. We labeled any submodel containing a part or whole representation of a composite model as an “Executive Submodel.” In addition, we attempted to categorize submodels further by their function and the outputs they produce. We found this somewhat difficult to do and think our current ad hoc submodel categories are shaky and that better methods of submodel categorization are needed.

## 5 CONCLUSION

In support of biomedical research and learning health systems, we offer a new method for CBK model composition that relies on decentralized web technology. Our method uses special digital objects called Knowledge Objects to establish operable pairs of web services and running computer‐processable functions. In this way, complex composite models are made accessible via the World Wide Web. As a feasibility demonstration, we connected 42 packaged submodels into a composite CBK model called CM‐IPP. We then used the CM‐IPP to compute individualized life‐gain estimates for a host of evidence‐based preventive services. By accessing CM‐IPP via a web service, client applications gain powerful computational and reasoning capabilities quickly and easily. Using this approach, CBK model‐makers can build network‐accessible composite models to advance research, education, and learning health systems.

## FUNDING INFORMATION

The Agency Healthcare Research and Quality sponsored this work through R21 grant number HS026257‐01. More information about this funded project is available in https://digital.ahrq.gov/sites/default/files/docs/citation/r21hs026257-firedman-final-report-2020.pdf.

## CONFLICT OF INTEREST

Dr. Taksler received consulting fees from the University of Michigan, Ann Arbor on a grant funded by the Agency for Healthcare Research and Quality (R21HS026257), directly related to the submitted work. The other authors assert they have no conflicts of interest.
